# Anti‐malarial drug effects on parasite dynamics in vivax malaria

**DOI:** 10.1186/s12936-021-03700-7

**Published:** 2021-03-21

**Authors:** Nicholas J. White

**Affiliations:** 1 Mahidol-Oxford Tropical Medicine Research Unit, Faculty of Tropical Medicine, Mahidol University, Bangkok, 10400 Thailand; 2 Centre for Tropical Medicine and Global Health, University of Oxford, Oxford, UK

## Abstract

Relapses of *Plasmodium vivax* malaria are prevented by 8-aminoquinolines. If hypnozoites survive, then the subsequent blood stage infections in early relapses (< 2 months) are suppressed by the slowly eliminated anti-malarial drugs used to treat the blood stage infection (chloroquine, artemisinin combination treatments), but they are not usually eliminated. The 8-aminoquinolines have significant blood stage activity which contributes to therapeutic responses. The latent interval from primary infection to early relapse depends on the number of activatable hypnozoites, the dose of anti-malarial, its pharmacokinetic properties, the level of resistance (minimum inhibitory concentration) and immunity. The dose–response relationship for radical curative efficacy of primaquine and tafenoquine is steep over the total dose range from 1.5 to 5 mg base/kg which may explain the poor efficacy of tafenoquine at the currently recommended dose.

## Background

Relapse of malaria refers to a recurrence of the infection derived from dormant liver stage parasites called hypnozoites. Relapses occur in infections with *Plasmodium vivax* and the two *Plasmodium ovale* sibling species, and in several primate malarias—notably *Plasmodium cynomolgi*, which is the usual animal model of vivax malaria. In tropical areas of Asia and South America, where *P. vivax* is often the dominant malaria parasite, relapses occur at frequent intervals. They are a major cause of morbidity, particularly in young children, and in areas of higher transmission, they contribute to anaemia and childhood mortality [1, 2].

Relapse is a major impediment to malaria elimination because the only drugs that can prevent them and thereby provide “radical cure” are the 8-aminoquinolines, and they are under used. This class of drugs causes oxidant haemolysis in patients with glucose-6-phosphate dehydrogenase deficiency (G6PDd) which is very common in tropical areas (X-linked; gene frequencies average 8–10% but can be as high as 35%) [3]. G6PD testing is often unavailable, so prescribers are commonly reluctant to risk haemolysis in order to prevent relapse.

**Fig. 1:**
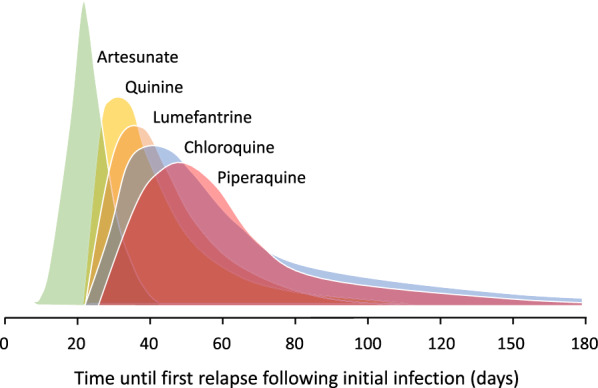
The temporal distribution of first relapses in tropical *P. vivax* infections treated with different anti-malarial drugs. Artesunate was given for 5 or 7 days. The quinine interval was derived from studies in which quinine was given for 14 days. The interval is shorter after currently recommended 7-day quinine regimens. The other drugs are given in 3 day regimens. Lumefantrine and piperaquine are the slowly eliminated components of artemisinin-based combinations. This temporal pattern of first relapses reflects the elimination kinetics of the different drugs and thus the duration of suppression of asexual stage multiplication in the relapsing infection [8–28]

Radical cure (to prevent relapses) needs to be used more widely, but questions remain on optimum dosing. Evaluating relapse prevention is compromised by the difficulty in differentiating recrudescence (recurrence of the blood stage infection), relapse and reinfection. This is because relapses can be with *P. vivax* parasites which are from the incident infection, and thus genetically identical or closely related, or can arise from previously acquired hypnozoites which are genetically unrelated to the incident infection [4, 5]. The reduction in the probability of relapse (radical cure efficacy) depends on the dose of 8-aminoquinoline (and the quality and bioavailability of the formulation) and the number of activatable hypnozoites in the liver (and thus the intensity of previous malaria exposure). Other important determinants are the degree of immunity and, for primaquine, the presence of loss-of function cytochrome P450 genetic polymorphisms. This is because cytochrome P450 (particularly 2D6) is responsible for primaquine bioactivation. The exact mode of action of the 8-aminoquinolines is not known but involves generation of reactive oxidative intermediates. Primaquine is metabolized rapidly (elimination half-life ~ 5 h) via monoamine oxidase to a biologically inert metabolite—carboxyprimaquine—and, through a separate pathway, to several active hydroxylated metabolites mainly via CYP 2D6 [6]. Based on studies of the gametocytocidal activity of primaquine in *P. falciparum* (which is a reasonable pharmacodynamic proxy for the hypnozoitocidal activity) the proposed sequence of events is as follows [7]. The unstable hydroxylated metabolites of primaquine are oxidized to quinoneimines generating local hydrogen peroxide—which is parasiticidal. The quinoneimines in turn are substrates for cytochrome P450 NADPH:oxidoreductase (POR or CPR) resulting in more hydrogen peroxide accumulation and augmenting the parasiticidal effect. Synergy between the 8-aminoquinoline and the concomitant 4-aminoquinoline (or structurally related compounds) used to treat the blood stage infection has also been shown to contribute to radical curative efficacy. Slowly eliminated anti-malarials used in the treatment of the acute malaria infection are also a major determinant of the interval to patent relapse as, following release of parasites from the liver into the blood approximately 2 weeks later, residual levels of these drugs reduce subsequent asexual blood stage multiplication (Fig. 1). This review assesses the effects of anti-malarial drugs on *P. vivax* parasite dynamics and therapeutic responses in early relapse.

### The latent period

**Fig. 2:**
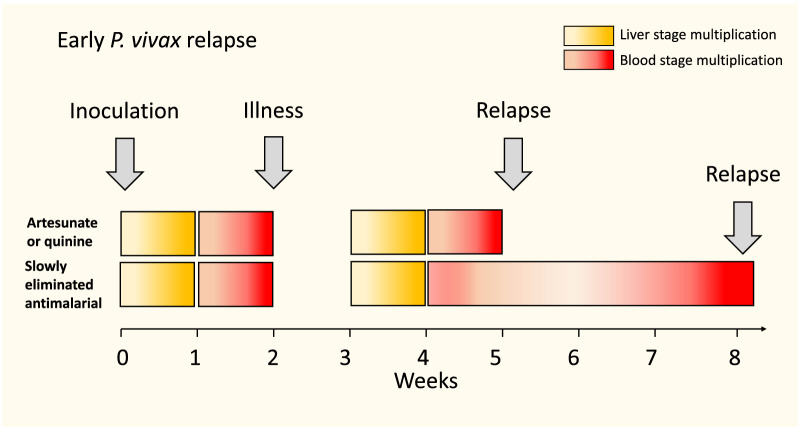
Usual intervals from initial sporozoite inoculation to primary illness and then first relapse in tropical *P. vivax.* This assumes multiplication in the developing hepatic schizonts is similar in the primary infection and relapse. Relapse patency is delayed after treatment with slowly eliminated anti-malarials because of blood stage multiplication inhibition from residual drug levels

This is the interval between the initial patent infection and the subsequent relapse (which is usually confirmed by microscopy). The latent period for the tropical, frequently relapsing, “strains” (represented by the “Chesson” strain) is approximately 3 weeks if a rapidly eliminated anti-malarial is used for primary treatment (i.e. artesunate or quinine) [29–31]. It is variably longer if slowly eliminated anti-malarial drugs are used for the reasons described above (i.e. suppression of blood stage multiplication; Fig. 1). In temperate regions *P. vivax* may have a long incubation period of 8–9 months or a similarly long interval between the primary illness and the relapse [31]. Long latency is an important consideration in assessing radical curative efficacy, but is unaffected by residual drug levels from the primary infection treatment. The 3-week latency for the frequent relapsing *P. vivax “*tropical strains” which predominate today suggests that their hypnozoites begin to divide approximately 1 week after the onset of the primary infection illness (i.e. 3 weeks after the preceding sporozoites were inoculated) (Fig. 2). This assumes that once the hypnozoite or hypnozoites awaken and intrahepatocyte cell division starts then the rate of multiplication within the developing schizonts is similar to that in the primary (incident) infection. Thus, the interval from acute illness to maturity of the hypnozoite derived relapse hepatic schizonts is approximately 2 weeks. The number of merozoites liberated into the blood by a single mature hepatic schizont of *P. vivax* is approximately 10,000 [32]. Once liberated, asexual multiplication of the blood stage infection in the absence of drugs or effective immunity is approximately 8–10 fold every ~ 48 h. As a result, in adults (blood volume approximately 5 L), if only one schizont has ruptured then patent parasitaemias (> 50/uL) are reached in four asexual cycles (8 days) [33]. This represents four multiplication steps from 10^4^ to 10^8^ asexual parasites. The interval to patency (microscopy detectable parasitaemia) shortens with smaller blood volumes (i.e. children) and larger numbers of activated hypnozoites (e.g. the interval would be 6 days if 10 hypnozoites were activated) [34–36]. The ~ 50/uL limit of detection for routine microscopy and the latest rapid diagnostic tests (RDTs) is close to the pyrogenic density in *P. vivax* infections [37, 38].

In summary the latent period depends on several factors which include.

- Blood stage immunity against the asexual parasites.
- Pyrogenic density.
- Body size and thus blood volume (i.e. age).
- Concentrations and inhibitory activity of blood stage anti-malarial drugs.
- The number of surviving activated hypnozoites.

### The hypnozoite burden

Much remains to be learned about hypnozoite biology, but clinical and experimental studies strongly suggest that in malaria endemic areas, individuals often have a few dormant, but *activatable*, hypnozoites in their livers [31]. If the proportion of acute falciparum malaria infections which are followed shortly afterwards by a *P. vivax* infection (with an interval corresponding to the latent period) is a guide, this suggests that between 20% and 60% of endemic area residents have pre-existing activatable hypnozoites [39–42]. This explains why relapses in endemic regions are often with *P. vivax* parasites which are genetically unrelated to those causing the index infection [4, 5, 31]. It may, therefore, be inferred that in endemic areas there is a quasi-steady state in which individuals acquire hypnozoites, usually with a blood stage infection (which may or may not be symptomatic), and later lose these hypnozoites by activation or hepatocyte apoptosis. The hypnozoite burden at any time depends on the previous intensity (force) of infection and the individual’s exposure to infectious mosquito bites. In the majority of *P. vivax* endemic areas outside Oceania the average entomological inoculation rate is low (~ 1 infectious bite per person per year) although, even in these low transmission settings, there are focal areas (“hot spots”) of much higher transmission [43]. It has been estimated that approximately half the inoculated sporozoites of “Chesson strain” *P. vivax* are destined to become hypnozoites [44]. As the median sporozoite inoculum from an infected mosquito bite is estimated to be 6–10 sporozoites, each inoculum would give a median of 3–5 hypnozoites [45–47].

Intense exposures in areas of high transmission (e.g. on the island of New Guinea) result in heavy hypnozoite burdens and, in non-immune visitors, these cause multiple symptomatic relapses [48, 49], but in most other areas of the world *P. vivax* transmission is much lower. There are also substantial geographic differences in the proportions of *P. vivax* infections which relapse. Overall, in endemic areas the majority of relapses are asymptomatic as older children and adults acquire disease controlling immunity (“premunition”) [50, 51]. In low transmission regions between 20% and 85% of patients presenting with acute vivax malaria (and not receiving radical treatment with primaquine) will have no detectable relapse of *P. vivax* [31, 52, 53]. This strongly suggests that the number of activatable hypnozoites is low (i.e. the individual probability that a hypnozoite can be activated is low) [31]. The more hypnozoites that are activated, the shorter is the latent period. This was demonstrated clearly in Leon Schmidt’s classic investigations of *P. cynomolgi*, the closest animal “model” of vivax malaria [36, 54–56]. There are two reasons for this: first, the starting number of merozoites liberated by the hepatic schizonts is larger, and second is because there is a distribution of latent periods, and it is the progeny of the first schizont to liberate merozoites, that will usually reach patency first [31].

### Blood stage multiplication

Blood stage multiplication of the relapsing infection is constrained by “immunity” (if there is any) to the relapsing parasites, host factors such G6PD deficiency, and by the blood concentrations and activity of anti-malarial drugs [56–58].

#### Chloroquine

**Fig. 3:**
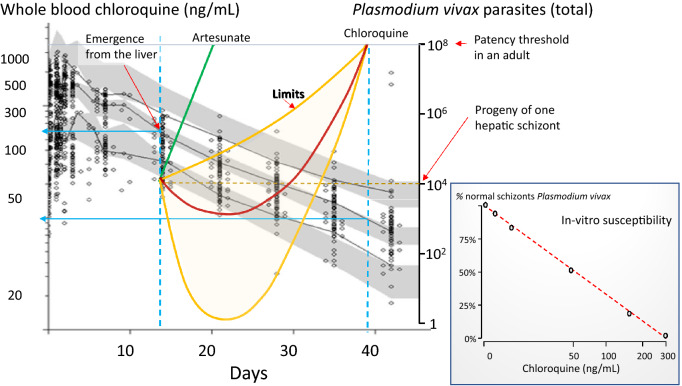
Whole blood chloroquine levels following the treatment of adults with acute vivax malaria (dose 25 mg base/kg) on the Thailand—Myanmar border. The three grey lines show the 95th, 50th and 5th percentiles for the observed data. The corresponding 95% confidence intervals from 1000 simulations based on the population pharmacokinetic model are shown as shaded areas. The black dots are the true observations; from Hoglund et al. [60]. The upper horizontal blue arrowed line shows the corresponding 50th percentile whole blood chloroquine concentrations at the approximate time of tropical *P. vivax* relapse liberation of merozoites into the circulation, and the lower blue arrowed line shows the corresponding approximate whole blood chloroquine concentration at relapse patency. The first relapse of *P. vivax* malaria emerges around 14 days after starting treatment (first vertical light-blue dashed line) at which time the whole blood chloroquine levels have fallen to around 150 ng/mL. If artesunate (5–7 days) had been the treatment there would have been no suppression of blood-stage multiplication of the relapse, as the drug would have been fully eliminated a week earlier. Relapses following artesunate would have become apparent around 3 weeks after starting treatment (green line). With the slowly eliminated chloroquine relapses are delayed by suppression of multiplication. The exact sub-microscopic profile is unknown but should be within the yellow lines (limits). Based on concentration-effect modelling by Watson et al. [58] the most likely parasitaemia-time profile is shown by the red line. The smaller graph on the right shows a chloroquine in*-*vitro susceptibility assessment by Russell et al. [65]

Chloroquine is the most widely used treatment of vivax malaria. Chloroquine has complex pharmacokinetic properties characterized by an enormous apparent volume of distribution and very slow elimination [59]. However, by 2 weeks after starting the 3-day anti-malarial treatment, when relapse merozoites first appear from the liver, whole blood chloroquine concentrations have fallen to ~ 150 ng/mL (although there is up to six-fold inter-individual variation) [59–61] (Fig. 3). These concentrations are still sufficient to inhibit growth of *P. vivax*—unless there is high level resistance. As a result, unless chloroquine concentrations in the blood are unusually low, asexual parasite densities of the first relapse will fall initially after emerging from the liver until the chloroquine concentrations fall to below the minimum inhibitory concentration (MIC). Thereafter the parasite densities rise again and will reach patency several weeks later (the first relapses after chloroquine treatment generally become patent around 6 to 7 weeks after the initial treatment—although there is significant inter-individual variation) [8–17, 57, 58, 61, 62] (Figs. 2 and 3). The interval to the first detected recurrence depends on the exposure of the drug (and thus the dose of chloroquine administered) [52, 58]. As a result, the first evidence of chloroquine resistance is less inhibition of blood stage growth and earlier patency of the first relapse [57]. For chloroquine, the appearance of relapses within 35 days of starting chloroquine together with a whole blood chloroquine + desethychloroquine (combined) concentration over 100 ng/mL has been proposed as an indicator of resistance [63, 64].

In summary, the first relapse following chloroquine treatment of vivax malaria in tropical areas has probably persisted in the blood for 3–5 weeks before becoming patent. In contrast, following treatment with rapidly eliminated drugs such as artesunate or quinine for 5–7 days, there is no suppression of asexual stage multiplication and the first relapse becomes patent approximately one week after leaving the liver [16, 18, 19, 30] (Figs. 2 and 3).

#### ACT partner drugs

**Fig. 4:**
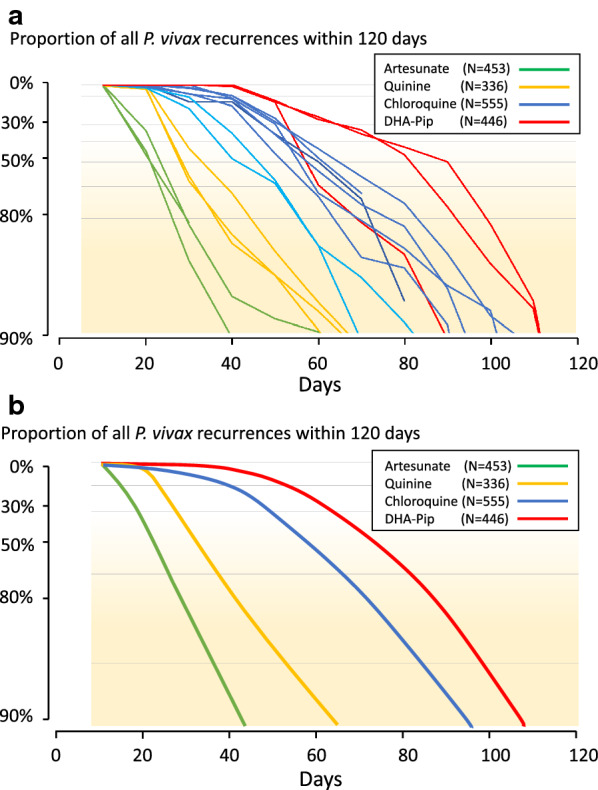
Recurrence rates following the treatment of acute *P. vivax* malaria in trials with different anti-malarial drugs, but without 8-aminoquinolines, in which there was frequent assessment [8–16, 18–22, 24, 25, 28, 30, 35, 61, 67–69]. The percentages are the proportions of all recurrences observed by 120 days over time. They show more rapid reappearance (presumed relapse) of vivax malaria with more rapidly eliminated drugs. Left (**a**): Individual trials (light blue shows two trials from the Thailand–Myanmar border where there is low grade chloroquine resistance, darker blue lines are from trials conducted in areas where *P. vivax* is generally more chloroquine sensitive). Right (**b**): average profiles

Lumefantrine is the most rapidly eliminated of the ACT partner drugs and so suppresses *P. vivax* relapses for a shorter period [14, 20, 21] than the more slowly eliminated piperaquine, mefloquine, desethylamodiaquine, pyronaridine or chloroquine [8–17, 20–27, 61, 62]. Cumulative relapse rates are only slightly lower with the slowly eliminated anti-malarials [16, 28, 31]. This suggests that few relapses are eliminated by the residual anti-malarial drug concentrations. Nevertheless, delaying relapse may still have some therapeutic advantages such as allowing more time for recovery from illness and anaemia [2, 22, 66]. It is important therefore to have sufficient follow-up to capture relapses when comparing two treatments with different elimination kinetics, otherwise the more slowly eliminated drug will appear to have superior recurrence preventing efficacy. For drugs such as chloroquine, desethylamodiaquine, piperaquine, mefloquine and pyronaridine, four months is probably the minimum duration of follow-up (Fig. 4).

### Radical cure

**Fig. 5:**
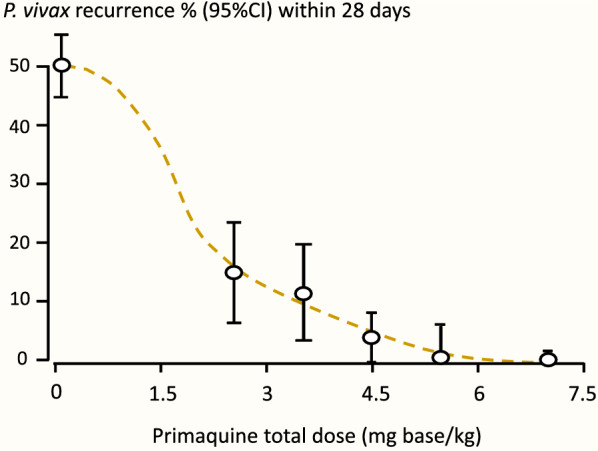
Recurrence rates (presumed relapses) in hospitalized Thai adults with acute vivax malaria who were treated with artesunate for 5 or 7 days and given different durations of primaquine at 30 mg (base)/day. From Pukrittayakamee et al. [72]

Relapses are prevented by the hypnozoitocidal effects of the 8-aminoquinolines [70, 71]. These are the only available drugs which kill hypnozoites [71]. Radical cure efficacy is dependent on the total dose administered [52, 71, 72] (Fig. 5) and the number of activatable hypnozoites.

A simple model is proposed in which the number of activatable hypnozoites follows a Poisson distribution with parameter λ (the mean number of activatable hypnozoites). If a drug exposure results in a probability **p** of “killing” each hypnozoite independently, then the probability of the binary outcome of whether relapse occurs (defined as the probability that at least one hypnozoite remains) or not increases as a function of the mean number of activatable hypnozoites (λ).

Under this Poisson-binomial model, the proportion of patients with at least 1 hypnozoite left after radical drug treatment.

$$\begin{array}{l} = {\text{proportion relapsing}}.\\ = 1 - ({\text{e}^{ - \uplambda }}/{\text{e}^{ - \uplambda \text{p} }}).\\ = 1 - {\text{e}^{ - \uplambda (1 - \text{p})}}. \end{array}$$

**Fig. 6:**
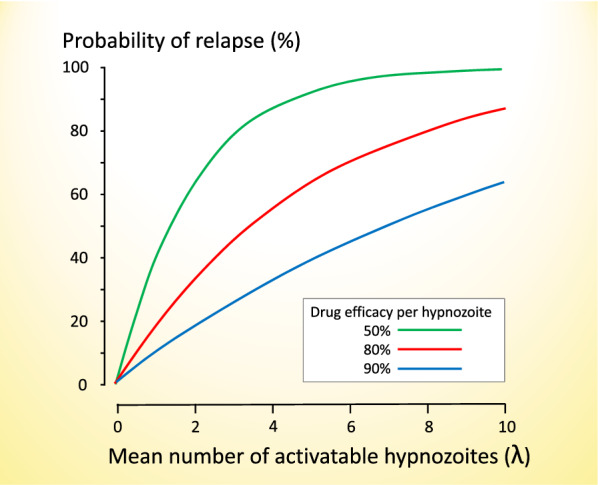
The relationship between the mean number of activatable hypnozoites ($\lambda )$ and the per-hypnozoite probability of being killed by the radical curative treatment. This is based on a simple model in which the number of activatable hypnozoites follows a Poisson distribution. If a drug exposure results in a probability **p** of “killing” each hypnozoite independently, then the probability of the binary outcome of whether relapse occurs or not increases as a function of the number of surviving activatable hypnozoites. The proportion of patients with at least one hypnozoite surviving (i.e. relapsing) = 1 – e^−λ (1−p)^

Figure 6 shows this relationship for different per hypnozoite drug efficacies and illustrates how the observed efficacy is strongly dependent on the latent hypnozoite load.

**Fig. 7:**
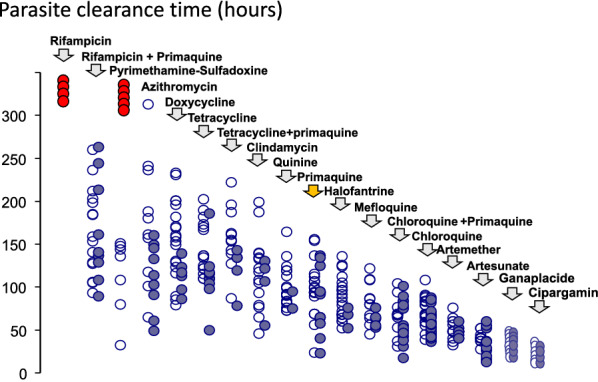
Parasite clearance times in adult Thai patients with vivax malaria after different anti-malarial drug treatments [82]. Parasite counts were measured at ≤ 6 h intervals on thin films, and at ≤ 12 h intervals on thick films. The *open circles* are individual asexual parasite clearance times, and the *closed circles* are corresponding gametocyte clearance times. The red circles represent failure to clear parasitaemia and administration of rescue treatment. Primaquine monotherapy is highlighted (yellow arrow)

If relapses do occur after a primaquine radical cure regimen has been given, then they are not markedly delayed. This suggests that subsequent hepatic schizont development is not retarded in the surviving hypnozoites. Thus, relapses which do occur following 8-aminoquinolines are likely to arise from few, and often a single hypnozoite (the exception being in individuals with CYP 2D6 loss of function polymorphisms who do not bioactivate the primaquine). There are some geographic differences in susceptibility. The long latency “strains” (such as the Korean *P. vivax* which gave the currently recommended dose regimens of primaquine) are more sensitive [73, 74] and “strains” from Oceania (Chesson type) are considered to be intrinsically more resistant [28–30, 75]. There is no evidence for acquired resistance in hypnozoitocidal activity [31]. However, acquired resistance to the asexual stage activity of these drugs has been demonstrated in clinical experiments [76]. The blood stage activity of primaquine against *P. vivax* is sufficient to cure the infection when given alone [72, 77]. The parasitological response to primaquine alone is slower than following treatment with chloroquine. Parasite clearance rates are similar to those observed with quinine treatment [56] (Fig. 7). This significant blood stage activity contributes to the overall blood stage therapeutic effect in combination with chloroquine or an ACT, and therefore reduces the probability of recrudescence (as well as the hypnozoitocidal effect reducing relapse). There is evidence for synergy between the 8-aminoquinolines and other quinoline anti-malarials in blood stage activity as well as in radical curative activity [78–80]. Highly chloroquine resistant *P. vivax* may still respond to chloroquine and primaquine because of primaquine’s blood stage activity [77, 81]. As described earlier, the similarity of the interval to first relapse, whether primaquine has been given or not, does suggest that hepatic schizont cell division is not affected in the progeny of the few hypnozoites that survive radical cure regimens.

### Assessing drug effects in vivax malaria

#### Initial response

The assessment of initial responses to drug treatment in vivax malaria is generally similar to that in falciparum malaria, but there are some important differences. First, parasite densities are lower in vivax malaria and very seldom exceed 100,000/uL [37]. Serial assessment of parasite densities to assess parasite clearance rates is therefore less accurate. Second, *P. vivax* does not cytoadhere appreciably (although it does accumulate markedly in the spleen [83]). This removes the major complexity of distinguishing drug effects from sequestration in assessing parasite density–time relationships. Third, persistent gametocytaemia is not a confounder as asexual stages of *P. vivax* are considered equally susceptible to the anti-malarial drugs [82] (Fig. 7). Thus, slowing of asexual and sexual stage clearance is a measure of drug resistance, and is a useful indicator of high-level resistance, but it is insensitive at lower levels of resistance. For slowly eliminated drugs, such as chloroquine, which have multiexponential elimination concentration profiles, the blood concentrations in the first few days of treatment are substantially higher than those 2 weeks later when relapse merozoites first emerge from the liver (Fig. 3). These relatively high concentrations retain full activity (E_max_) against moderately resistant *P. vivax*. Taylor et al. studied chloroquine resistant *P. vivax* in Papua over 20 years ago; despite a 71% treatment failure rate within 28 days, the mean (95% CI) parasite clearance time was 2.4 days (1.9 to 2.9 days) i.e. similar to that elsewhere in sensitive *P. vivax* infections [67]. Thus, slowing of parasite clearance for chloroquine (and for other drugs with similar pharmacokinetic properties) is an insensitive measure of developing resistance.

#### Radical cure

**Fig. 8:**
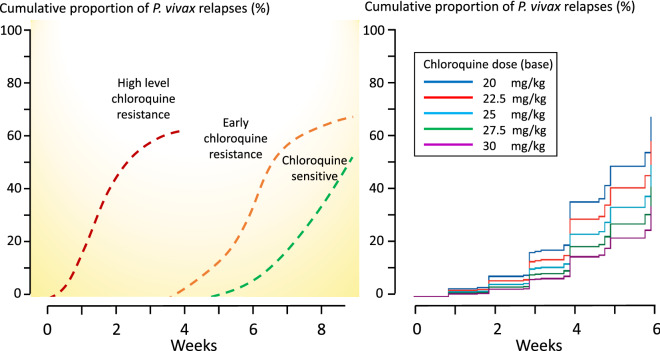
Left: Temporal profiles of *P. vivax* recurrence at different levels of chloroquine resistance. At high levels of resistance (as seen currently in Indonesia and Papua New Guinea) early recrudescence dominates (red dashed line). At lower levels of resistance (as seen currently on the Thailand–Myanmar border) the relapses appear earlier than previously, and a few appear within 28 days (orange dashed line). In fully sensitive infections no relapses appear within 4 weeks, and very few occur before 6 weeks (green dashed line). Right: The effect of chloroquine dose (and thus exposure) on the time to *P. vivax* recurrence; from Commons et al. [52]

Unless there is such high-grade resistance that total asexual parasite burdens 2 weeks after starting treatment (e.g. with chloroquine) exceed 10,000 (the approximate number of merozoite progeny of one activated hypnozoite) then relapses will generally precede recrudescences (if there are any). High level chloroquine resistance, where recrudescence predominates, is seen currently only on the island of Borneo, in Indonesia and in Oceania [25, 63, 64, 67–69] (Fig. 8). The clinical manifestation of worsening chloroquine resistance will therefore be earlier and earlier recurrences (with an increasing proportion of parasites being genetically similar to the primary infection as recrudescences comprise an increasing proportion of recurrences). Even if the recurrence is caused by a new infection, it still reflects resistance as only resistant parasites can grow to reach patent parasitaemias through residual drug concentrations (e.g. 2–4 weeks after chloroquine treatment).

**Fig. 9:**
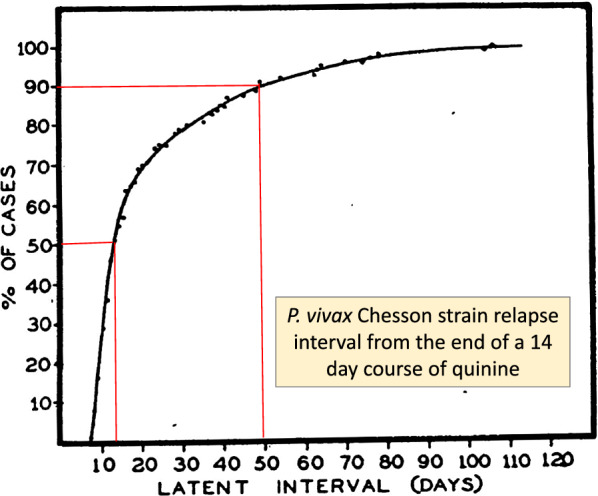
Cumulative proportion of *P. vivax* relapses in volunteers infected with Chesson strain *P. vivax* from Alving et al. during evaluations of different 8-aminoquinolines for radical cure [29]. The latent interval was calculated from the end of a 14-day course of quinine (2 g/day of either the sulfate or hydrochloride salts in six divided doses) to treat the primary infection

Most published clinical trials describing radical curative efficacy report cumulative recurrence rates after different drug regimens (Fig. 9). As explained, drug exposures, parasite numbers and levels of resistance all contribute to the interval to relapse. The first relapses in clinical trials will therefore be in patients with higher numbers of hepatic schizonts, more resistant parasites (referring to blood stage resistance) and lower drug levels. The net result is a skewed distribution of intervals to relapse, with later modal intervals following treatments with more slowly eliminated anti-malarials (Fig. 1). If patients or volunteers are studied outside the malaria transmission area then reinfection is not possible, so recurrences must be either relapses or recrudescences. Within the endemic area reinfection occurs and the proportion of recurrences which are not relapses (i.e. reinfections) rises with time, and is strongly influenced by the rainy season in low transmission settings. With longer follow-up recurrent infections comprise an increasing proportion of cases. This dilutes the end point in comparative trials. The progressive loss of specificity reduces statistical power and compromises non-inferiority comparisons. The more intense the transmission (reinfection probability), the more rapid is the dilution. Time-to-event information and parasite genotyping allows a probabilistic discrimination between relapse and reinfection which improves the power and precision of therapeutic assessments [84]. However, occasional late relapses (many months later) do still occur, even with frequent relapsing *P. vivax*, as Coatney showed unequivocally in volunteers who received single infected mosquito bites [35]. But the large majority of recurrences which occur within a few months are relapses, even in higher transmission settings,—and these are prevented by sufficient doses of 8-aminoquinolines [71, 80]. What then is the optimum duration of follow-up to assess radical cure efficacy? This depends first on whether long-latency *P. vivax* is prevalent (in which case 1 year is minimum), and then on the drugs used. In general, the majority of early relapses occur within 2 months, most have occurred within 4 months, and there are relatively few incident relapses thereafter. Thus, 6 months is a reasonably conservative duration of follow-up for chemotherapy studies of frequent relapse *P. vivax* and 4 months will still provide the majority of comparative information.

The radical curative efficacy of primaquine has been reviewed extensively [70]. The total dose of 3.5 mg base/kg (i.e. 0.25 mg/kg/day for 14 days or 0.5 mg/kg/day for 7 days) provides good efficacy everywhere except in South-East Asia and Oceania where a higher dose is needed (i.e. 0.5 mg/kg/day for 14 days or 1 mg/kg/day for 7 days). Despite this, most countries in these regions still recommend the lower dose. Recommended dosing in children has been the same as for adults, but exposures are lower in children (as is often the case for anti-malarial drugs) [85] so dose regimens might well need revising upwards in younger children. In patients who are G6PD deficient a weekly dose of 0.75 mg base/kg given for 8 weeks is recommended [71]. There are relatively few data to support the efficacy of this regimen and it is interesting to consider how it might work. By the time the first relapse merozoites leave the liver (~ 14 days) 3 doses (total 2.25 mg/kg has been taken). The first two doses (i.e. 1.5 mg/kg) act on the pre-erythrocytic stage but, by extrapolation from the 8-aminoquinoline dose-finding studies, this is expected to have a partial effect at most on hypnozoite development. Thereafter, the subsequent six doses might well suppress the blood stage infection, but how they prevent relapse is unclear. G6PD deficiency itself protects against vivax malaria, so it is quite possible that the iatrogenic oxidant activity that leads to haemolysis also potentiates the blood stage anti-malarial effect. Thus, overall relapse preventive efficacy could be higher in G6PD deficient than in G6PD normal patients. More information is needed on the efficacy of this regimen as some countries have recommended the once weekly primaquine dose for all patients (because G6PD testing is unavailable).

#### Assessing tafenoquine

Tafenoquine (WR 238605; etaquine) is a synthetic analogue of primaquine ([2, 6-methoxy-4-methyl-5-(3-trifluoromethylphenoxy) primaquine, succinate] which is eliminated slowly. Tafenoquine was developed to provide comparable anti-malarial activity to primaquine, but over a much longer time period so that it could be given in a single dose [86–88]. In other words, it was designed to be more stable than primaquine.

Although tafenoquine has asexual stage activity in-vitro, the concentrations required for an anti-malarial effect are high by comparison with in-vivo exposures [65, 89, 90]. This suggests that tafenoquine requires in-vivo bioactivation, as does primaquine, although this is debated. Interestingly the CYP 2D6 intermediate loss of function polymorphisms, which are associated with impaired radical curative activity of primaquine, do not seem to affect the efficacy of tafenoquine—although the numbers studied were small [91, 92]. This may reflect a large difference in activation rates as the rapidly metabolized primaquine has a shorter period in-vivo for each dose in which to generate bioactive intermediates. Assessing liver stage activities ex-vivo in cultured hepatocytes is more representative of in-vivo activity as the cultured hepatocytes can bioactivate the 8-aminoquinolines [93, 94]. The slow elimination of tafenoquine means that, if relapses do still occur, then the blood stage multiplication of the first relapse is reduced as, on emergence from the liver, it encounters suppressive concentrations of tafenoquine. This delays the time to relapse patency [95–97]. Thus, relapses which follow tafenoquine are delayed both because hypnozoite numbers are reduced and, for the progeny of those hypnozoites which survive, subsequent asexual stage multiplication is inhibited by the slowly eliminated anti-malarials. Tafenoquine and chloroquine are synergistic both in liver and blood stage activities, but the degree to which this contributes to overall efficacy is uncertain.

**Fig. 10:**
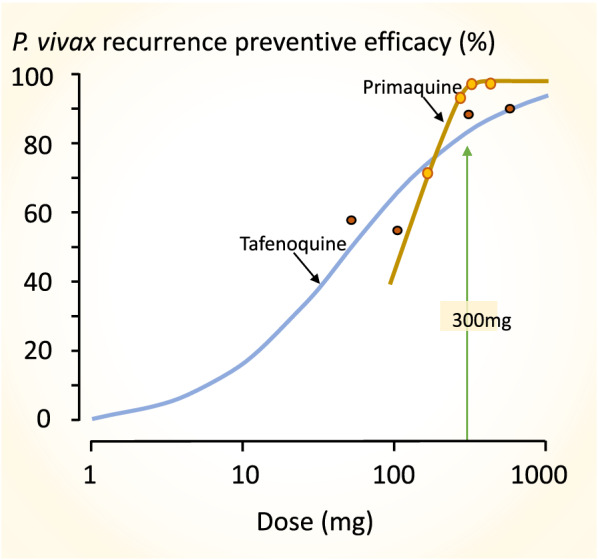
The approximate tafenoquine dose–response relationship (blue line) for prevention of *P. vivax* recurrence (180 days assessment) fitted by logistic regression to the data from the multinational DETECTIVE study; Llanos-Cuentas et al. [98]. Different doses of tafenoquine were given together with a standard 25 mg base/kg dose of chloroquine. As both relapse and recurrent infections are included, the true tafenoquine radical cure dose–response relationship has a steeper slope. The brown line shows the primaquine radical cure dose–response relationship (28 days assessment) from the study of different durations of primaquine in Thai adults with vivax malaria treated with artesunate shown in Fig. 5 [72]. Follow up for 28 days is insufficient to capture all relapses following artesunate so the true efficacy is slightly lower than portrayed

**Fig. 11:**
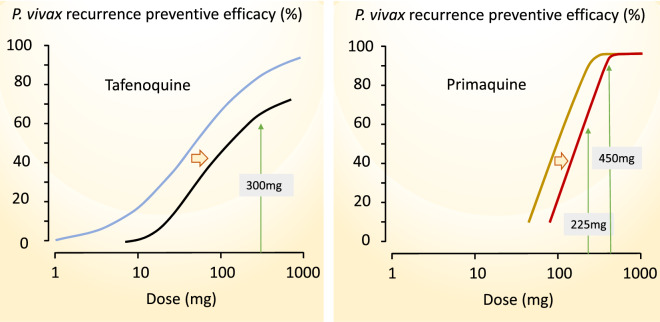
(Left): An illustration of how shifts to the right in the dose- response relationship (such as would be associated with higher hypnozoite loads) are expected to have a significant effect on the radical curative efficacy of the tafenoquine 300 mg adult dose (from Fig. 10). (Right): A similar illustration of the approximate dose–response relationship for primaquine in Southeast Asian *P. vivax* (from Fig. 10). This indicates that right shifts would give a greater reduction in radical curative efficacy of the widely used primaquine adult total dose of 210 mg (15 mg base/day for 2 weeks or 30 mg/day for 1 week), than they would on the WHO recommended (but rarely followed) 420 mg adult total dose (30 mg base/day for 2 weeks or 60 mg/day for 1 week)

Dose finding studies for tafenoquine show a clear dose–response relationship up to single adult doses of 600 mg [98]. In phase 3 pre-registration trials the currently recommended adult dose of 300 mg performed poorly in South-East Asia. It was significantly inferior in recurrence prevention compared with a low dose of primaquine (15 mg adults dose/day), and cure rates in South America were also disappointing [97, 99, 100]. The dose-finding study summarized in Fig. 10 indicates a relatively steep dose-response relationship over the adult dose range between 100 and 300 mg. Any shift to the right, such as would be associated with heavier patients, or larger hypnozoite burdens, receiving the fixed 300 mg dose (as shown in Fig. 5), would be expected to reduce radical curative efficacy significantly (Fig. 11).

## Conclusions

Slowly eliminated anti-malarial drugs delay, but usually do not eliminate *Plasmodium vivax* relapses. The blood stage activity of tafenoquine contributes to a significant delay in relapses becoming patent. The probability of relapse depends on many factors including the number of activatable hypnozoites in the liver and the exposure to, and activity of, the 8-aminoquinoline radical cure treatment. Dose–response assessments indicate that the total dose of primaquine currently recommended by National malaria control programmes in most countries of South-East Asia and Oceania (3.5 mg base/kg) is too low. While this dose might give high efficacy in patients with low hypnozoite burdens, it is not expected to give optimal cure rates in patients with heavier burdens. Clinical trials also indicate that the currently recommended dose of tafenoquine (300 mg) is too low. Higher doses of tafenoquine should be evaluated.

## Data Availability

All data are from published studies.
